# Triphenylamine-Based Helical Polymer for Flexible Memristors

**DOI:** 10.3390/biomimetics8050391

**Published:** 2023-08-26

**Authors:** Jinyong Li, Minglei Gong, Xiaoyang Wang, Fei Fan, Bin Zhang

**Affiliations:** 1 Key Laboratory for Advanced Materials and Joint International Research Laboratory of Precision Chemistry and Molecular Engineering, School of Chemistry and Molecular Engineering, East China University of Science and Technology, Shanghai 200237, China; 2 Shanghai i-Reader Biotech Co., Ltd., Shanghai 201100, China; 3 Guangxi Key Laboratory of Information Material, Engineering Research Center of Electronic Information Materials and Devices, School of Material Science and Engineering, Guilin University of Electronic Technology, Guilin 541200, China

**Keywords:** helical polymer, memristor, triphenylamine, synaptic function

## Abstract

Flexible nonvolatile memristors have potential applications in wearable devices. In this work, a helical polymer, poly (N, N-diphenylanline isocyanide) (PPIC), was synthesized as the active layer, and flexible electronic devices with an Al/PPIC/ITO architecture were prepared on a polyethylene terephthalate (PET) substrate. The device showed typical nonvolatile rewritable memristor characteristics. The high-molecular-weight helical structure stabilized the active layer under different bending degrees, bending times, and number of bending cycles. The memristor was further employed to simulate the information transmission capability of neural fibers, providing new perspectives for the development of flexible wearable memristors and biomimetic neural synapses. This demonstration highlights the promising possibilities for the advancement of artificial intelligence skin and intelligent flexible robots in the future.

## 1. Introduction

Due to increasing demands for flexible wearable electronic devices, flexible memristors that are eco-friendly and low-cost must be constructed [1,2,3,4,5,6]. Lightweight and high-performance flexible memristors have the potential to enable wearable devices with integrated information storage and processing capabilities, leading to innovative advancements in intelligent systems [7,8,9,10,11]. A variety of functional materials have been applied for flexible memristor applications, including organic–inorganic hybrid materials, organic polymers, and crystalline materials [12,13,14,15,16,17]. However, considerable efforts are still needed to achieve breakthroughs in parameters such as storage density, processing speed, and manufacturing costs for flexible memristors. In previous studies, nanostructured molecules have been used to fabricate various flexible electronic devices [18,19,20,21,22]. Shen et al. developed an artificial device that can replicate the function of the detrusor reflex arc in the biological bladder. The team successfully translated the stretching signal into control signals, which in turn activated electrochemical actuators for reflex control [23]. Zhang et al. fabricated a wearable 3D artificial neural network consisting of a three-layer cross-point array of Pt/HfAlO_x_/TaN memristors. They further simulated information transmission and processing resembling the human brain and successfully developed an artificial neural network for recognizing MNIST patterns. The network achieved an impressive accuracy of 88.8% [24]. Among these numerous candidate materials, nanowires have attracted attention, but memristors based on nanowires have rarely been reported. During the construction of nanowires, triphenylamine (TPA) derivatives and TPA-based polymers are attractive substances due to their conjugated structures and electrical conductivity [25,26]. TPA-based conjugated polymers have strong π–π interactions, which may enhance their charge transport characteristics [27,28].

Therefore, in this work, we polymerized polyisocyanide and triphenylamine to obtain organic helical nanowires containing triphenylamine groups. As illustrated in Table 1, our fabricated device, despite being a flexible memristor, displays electrical characteristics that are comparable with conventional semiconductor memristors. These findings suggest that our device is well-suited for data storage applications. The fabricated flexible memristor with an Al/PPIC/ITO-coated polydimethylsiloxane structure showed typical nonvolatile rewritable memory performance with an ON/OFF current ratio > 10^3^, a turn-on voltage of −0.72 V, and a turn-off voltage of 1.79 V. By utilizing the fibrous structure of the helical polymer, we also simulated the biological synapse function and brain nerve processing of information and associative learning process, which realized the basic functions of synaptic bionic devices. Double-pulse facilitation and inhibition, spike rate-dependent plasticity (SRDP), and empirical learning were used to study the information perception, transmission characteristics, and stability of the device. The pulse test results showed that the device met the basic requirements of a neural network for processing spatiotemporal information, providing a reference for applying memristors in brain-mimetic chips.

## 2. Materials and Methods

### 2.1. Materials

Unless specified otherwise, all the chemicals were purchased from Adamas and used without further treatment. Organic solvents were purified, dried, and distilled under dry nitrogen. All reactions were carried out under nitrogen atmosphere by a standard Schlenk technique. The PET substrate was coated with ITO (200 nm) on its surface, purchased from South China Xiangcheng Technology, had a square resistance ≤ 6 Ω, and was cut into 3 × 3 cm squares before use.

### 2.2. Measurements and Instrument

^1^H NMR was measured by a Bruker 400 MHz/AVANCE Ⅲ 400 spectrometer. UV-Vis absorption spectra were measured by Shimadzu UV-2540 spectrophotometer. FT-IR spectra were measured by a Spectrum 100 infrared spectrometer. AFM images were obtained by a Solver P47-PRO(NT-MDT Co) microscope; the Cyclic voltammetry and AC impedance curves were measured by a CHI-650D electrochemical workstation. The electrolyte solution was a 0.1 mol/L acetonitrile solution of tetrabutylammonium hexa-fluorophosphate. The three electrodes system was operated: Pt (working electrode), Ag/AgCl (reference electrode), and platinum wire (counter electrode).

### 2.3. Synthesis of 4-isocyano-N, N-diphenylanline (ICP)

4-Aminotriphenylamine (1.30 g, 5 mmol) was dissolved in 10 mL CH_2_Cl_2_ in a Schlenk tube. Then, chloroform (407 μL, 5 mmol), tetrabutylammonium hydroxide (26 mg, 0.1 mmol), and 10 mL 50% aqueous sodium hydroxide were added. The mixture was heated to reflux for 3 h, then cooled, washed with distilled water, and dried over anhydrous MgSO_4_. After the drying agent was filtered off and the solvent was removed, the residue was purified by column chromatography (Al_2_O_3_, petroleum ether/EtOAc 30:1). Yield: 0.40 g (1.5 mmol, 29%). ^1^H NMR (400 MHz, CDCl_3_): δ 7.42 (d, *J* = 8.7 Hz, 1H), 7.33 (t, *J* = 7.8 Hz, 2H), 7.16 (dd, *J* = 10.4, 8.1 Hz, 3H), 6.96 (d, *J* = 8.8 Hz, 1H).

### 2.4. Synthesis of Poly(N, N-diphenylanline isocyanide) (PPIC)

4-Isocyano-*N*, *N*-diphenylanline (0.13 g, 0.5 mmol) was dissolved in dry CH_2_Cl_2_ (2.25 mL). A solution of NiCl_2·_6H_2_O (1.066 mg, 0.0045 mmol) in MeOH (0.1 mL) was added via syringe, and the mixture was stirred at room temperature for 2 d. The reaction solution was first concentrated and then added dropwise to stirred methanol (10 mL). After filtration, the collected dark brown solid was washed several times with methanol and dried under vacuum at 40 °C overnight. Yield: 0.12 g (92%). GPC (THF): *M*_n_ = 3.15 × 10^4^, *PDI* = 1.36. ^1^H NMR (400 MHz, CDCl_3_): δ 7.42 (broad, CH, 2H), 7.00–7.25 (broad, CH, 12H).

### 2.5. Preparation of Al/PPIC/ITO Device

The ITO-coated PET was pre-cleaned by ultrasonication for 15 min each in deionized water, acetone, and isopropyl alcohol, in that order. The toluene solution of PPIC (10 mgmL^−1^) was spin-coated onto the ITO substrate at a spinning speed of 800 rpm for 10 s and then 2000 rpm for 30 s, followed by the removal of the solvent under vacuum at 60 °C overnight. Finally, the Al top electrode was thermally deposited on the polymer film by using a shadow mask under ultra-high vacuum. Ultrapure aluminum was used as the top electrode and deposited on the surface of the active layer by electron beam evaporation to obtain an Al/PPIC/ITO device with a sandwich structure. The vacuum degree of the evaporation process was 10^−7^ Torr. The radius of the electrode was 0.2 mm, and the thickness was 200 nm.

## 3. Results and Discussion

The helical polymer PPIC was prepared by the living polymerization of 4-isocyano-*N*, *N*-diphenylanline, and its structure was characterized. The atomic force microscopy (AFM) image in Figure S1 shows the helical, fibrous morphology of the polymer, which is similar to human nerve fibers. Each carbon atom on the polyisocyano main chain contained a bulky triphenylamine side chain that may have provided enough steric hindrance to stabilize the helical conformation [33]. The polymerization of PPIC was further characterized by Fourier Transform Infrared Spectroscopy (FTIR). As shown in Figure S2, the 2121 cm^−1^ peak attributed to the N≡C stretching vibration in the infrared spectrum disappeared in the spectrum of PPIC. This indicated that the N=C group was consumed during the polymerization to form PPIC. These results indicate that we successfully prepared helical PPIC.

**Figure 1:**
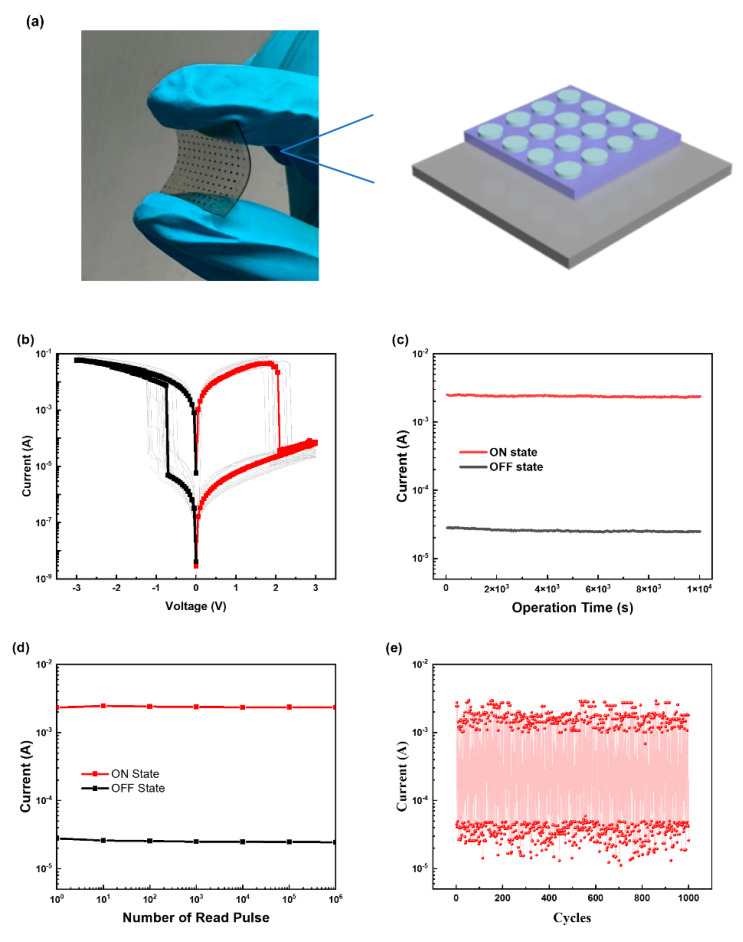
(**a**) Digital photo of the as-fabricated flexible memory device; (**b**) current–voltage characteristic of the Al/PPIC/ITO–coated device; (**c**) ON and OFF state currents as a function of the operating time under a constant stress of 1.0 V; (**d**) effect of read pulses of 1.0 V on the ON and OFF state currents of the device; (**e**) device switching stability under switching pulses with a pulse amplitude = ±3 V, pulse width = 10 μs, and pulse period = 20 μs.

To investigate the memory performance of PPIC, we fabricated a flexible resistive memory device with an Al/PPIC/ITO structure (Figure 1a) on an ITO-coated PET substrate. Through the FE–SEM test, we found that the thickness of the prepared film is 44.4 nm (Figure S3). As shown in Figure 1b, when a forward voltage sweep that ranged from 0 V to −3 V was applied to the device, the conductivity of the device changed from a low-conductivity state at a threshold voltage of −0.72 V (the OFF state; the device current at this time = 4.76 × 10^−6^ A) to a high-conductivity state (the ON state; the device current at this time: 6.18 × 10^−3^ A). This is the “writing” process of the device, which is very similar to the process occurring in digital information storage devices. During the next second forward scan, or when the power was turned off, the device remained in a high-conductivity state, indicating that the device has nonvolatile storage characteristics. In other words, the information stored in the device could be saved even without a power supply. When a reverse voltage from 0 V to 3 V was applied to the device, the current dropped rapidly from 2.8 × 10^−2^ A to 4.74 × 10^−7^ A at 1.79 V, which means that the device returned from the high-conductivity state to the low-conductivity state. This process is equivalent to the “erasing” of information. At this time, a negative voltage scan from 0 V to −3 V was applied to the device, which remained in a low-conductivity state, while a positive voltage scan turned the device back on. The above results show that the device had nonvolatile rewritable storage characteristics, while the current switch ratio reached as high as 10^3^. Figure 1c,d shows that the ON and OFF states of the device remained stable for 10^4^ consecutive seconds and 10^6^ consecutive readings. The cycling durability experiments also showed (Figure 1e) that the ON and OFF states remained unchanged even after 1000 switching cycles. Then, we carried out thermodynamic and optical characterization of the device (Figure S4).

**Figure 2:**
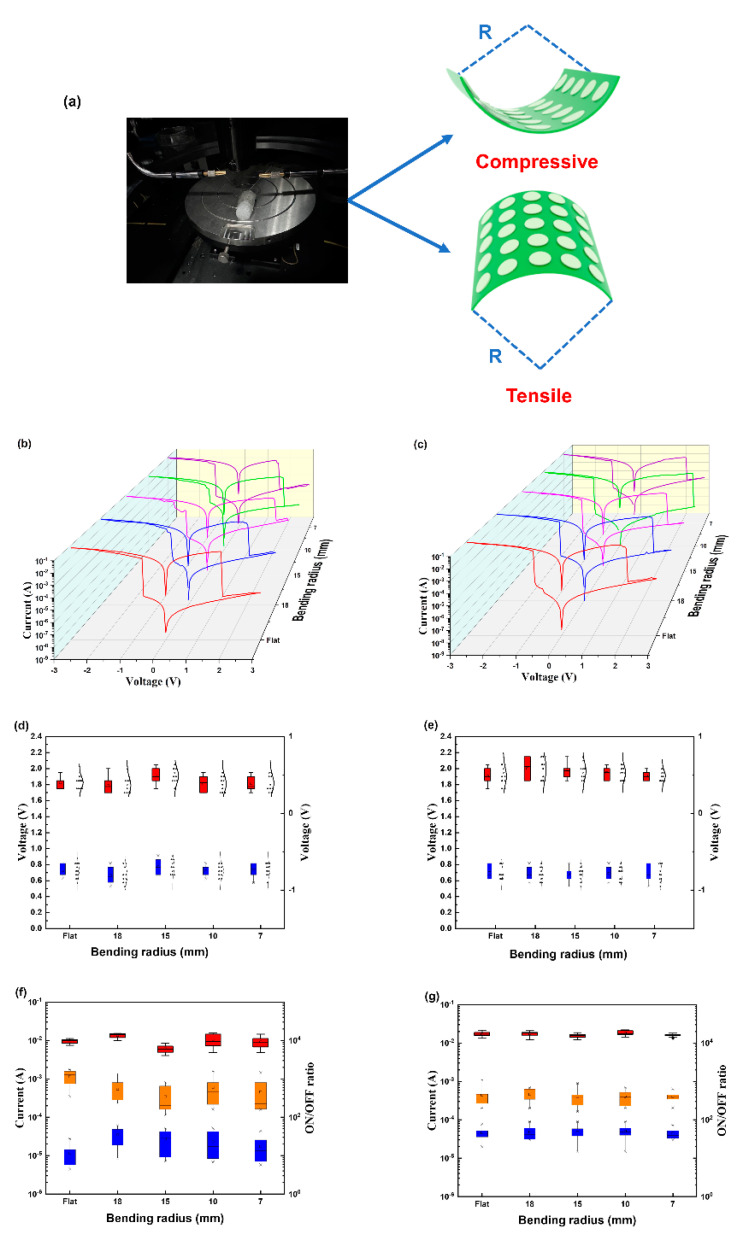
(**a**) Model diagrams of the fabricated flexible device in the bent state; (**b**,**c**) *I*–*V* characteristic curves of Al/PPIC/ITO flexible devices in different bending states (3b: under compressive strain, 3c: under tensile strain; different colors represent different bending states); (**d**,**e**) ON/OFF voltage distribution under different bending states; (**f**,**g**) ON/OFF state current of the device under different bending states and switch ratio distributions; ((**d**,**f**): compressive state, (**e**,**g**): tension state).

Mechanical stress changes the memory performance of electronic components, especially flexible electronic devices [34,35,36,37]. We speculated that the helical structure would remain stable in the bent state, so we tested the flexibility of memory devices made of helical PPIC by bending them from the flat state to distances of 7, 10, 16, and 18 mm. As shown in Figure 2b,c, the *I*–*V* characteristic curves show that the performance of the device under compressive and tensile strains was nearly unchanged, indicating that bending did not change the memory characteristics of the device. We also plotted statistical distributions of device performance parameters (ON/OFF state currents (*I*_ON_ and *I*_OFF_), threshold voltages (*V*_th(set)_ and *V*_th(reset)_), and ON-OFF ratios) in various bending states. The data in Figure 2d–g show that the above performance parameters of the device were nearly unaffected by the bending degree.

**Figure 3:**
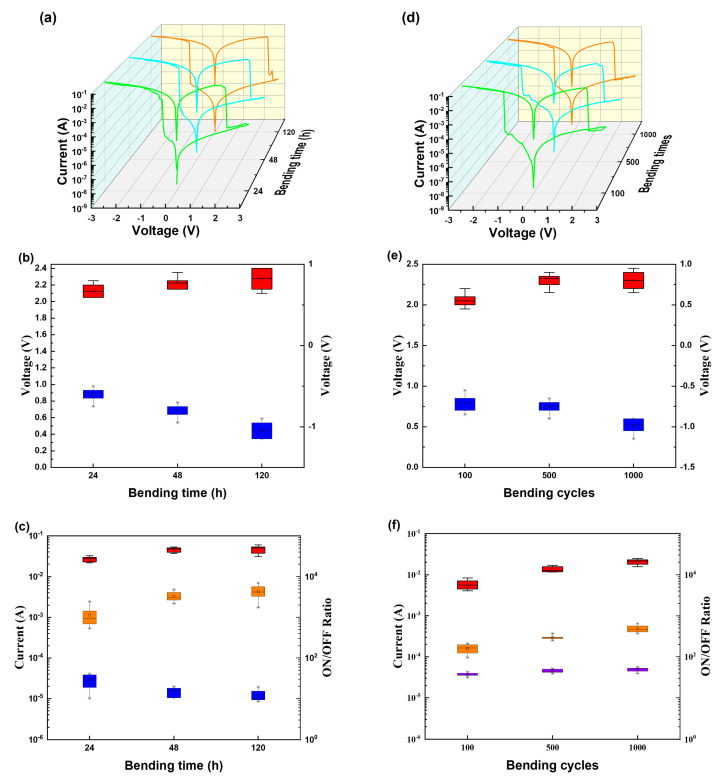
(**a**) *I*–*V* characteristic curves of Al/PPIC/ITO flexible devices at different bending times; (**b**) ON/OFF voltage distribution under different bending times; (c) ON/OFF state current of the device under different bending times and switch ratio distributions; (**d**) *I*–*V* characteristic curves of Al/PPIC/ITO flexible devices for different bending cycles; (**e**) ON/OFF voltage distribution under different bending cycles; (**f**) ON/OFF state current of the device under different bending cycles and switch ratio distributions.

After testing the different bending curvature radii of the device, we also investigated the effect of bending time and bending cycles of the device. As shown in Figure 3a–c, we bent the device by 10 mm and kept it for 24, 48, and 120 h, respectively. The storage performance under various bending times was good, and the ON-state voltage range was −0.5~−1.15 V. The OFF-voltage range was 2.05–2.4 V. As shown in Figure 3d–f, we bent the manufactured flexible devices 100, 500, and 1000 times while maintaining a bending degree of 10 mm. As the number of bending cycles increased, the device performance remained stable. The ON-state distribution was between −0.55~−1 V, and the OFF-state voltage was in the range of 1.95–2.45 V. These results show that helical polymer maintained a stable and flexible structure under various bending conditions (Figure S5), which means that PPIC is an ideal material candidate for preparing flexible rewritable memory devices.

**Figure 4:**
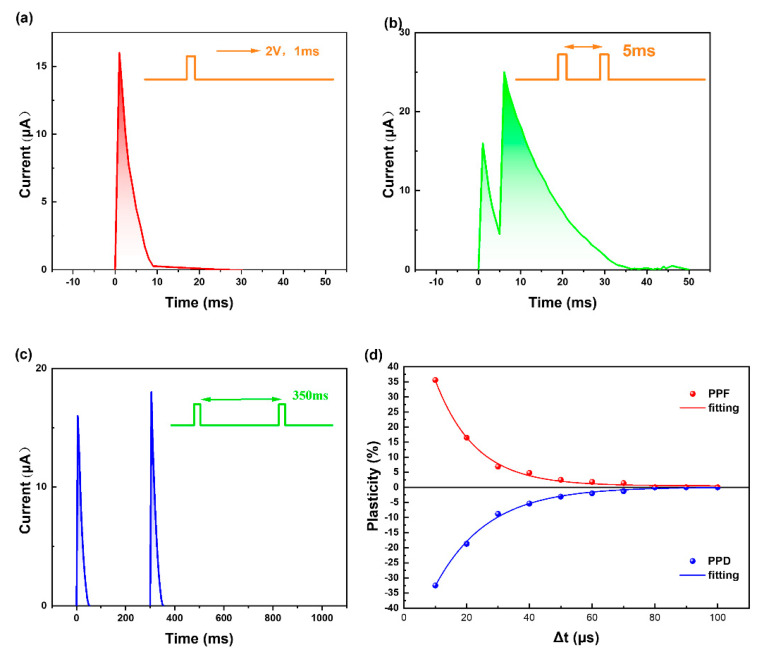
Pulse voltage measurement of memristor: (**a**) EPSC with a single positive pulse applied; (**b**) EPSC of PPF; (**c**) two positive pulses applied with an interval of 350 ms; (**d**) paired−pulse facilitation (PPF) and paired-pulse depression (PPD) index change with the time interval (Δ*t*) of the double−input presynaptic pulses (2 V and −2 V respectively, 10 μs).

After exploring its resistive memory performance, we next tested the memristor performance of the device. Most cranial nerves transmit information in the form of impulse waves. Only when the current neuronal signal reaches a certain intensity will the neural synapse sense and transmit to the next neuron, and impulses with a smaller intensity are regarded as noise [38,39]. The pulse test results of the bionic memristor are shown in Figure 4. When the input signal was a pulsed wave with an amplitude of 2 V and width of 1 ms, the excitatory post-synaptic current (EPSC) of the device was 15.93 μA. Applying a pulse in 5 ms intervals caused paired-pulse facilitation (PPF); that is, the EPSC caused by the second pulse was higher than that caused by the first pulse and reached 24.94 μA. This phenomenon is generally thought to result from the release of additional synaptic vesicles from residual calcium ions in human cells during the first neurostimulation signal [40,41]. When applied, only EPSC exceeding the threshold will form an effective output; that is, information can be transmitted from anterior neurons to posterior neurons via neural synapses. This can eliminate interfering factors during information transmission and is more similar to the working mode of the nervous system. When the interval between two pulses was increased to 350 ms, two EPSCs with an amplitude of 17.30 μA were detected, but no PPF phenomenon was observed, and no effective output was formed. This shows that when the pulse amplitude was fixed, the PPF phenomenon was more dependent on the pulse frequency. It was difficult for the pulse signal with an insufficient frequency to cause strong EPSC to form an effective output of the device. When we applied the same negative pulse voltage, the device showed paired-pulse depression (PPD). Biologically, PPD is thought to be caused by voltage-dependent inactivation of calcium channels or the temporary depletion of neurotransmitter vesicles accumulated in presynaptic neurons [42,43]. The PPF/PPD of bionic memristors was realized by controlling the voltage and frequency of positive and negative pulses. The facilitation ratio and inhibition ratio follow the relationship (*A*_2_ − *A*_1_)/*A*_1_ × 100% between the amplitude *A*_1_ produced by the first pulse and the amplitude *A*_2_ of the second pulse. This is usually only related to the pulse frequency, which prevents excessive pulse intervals from achieving double pulse facilitation or inhibition [44].

**Figure 5:**
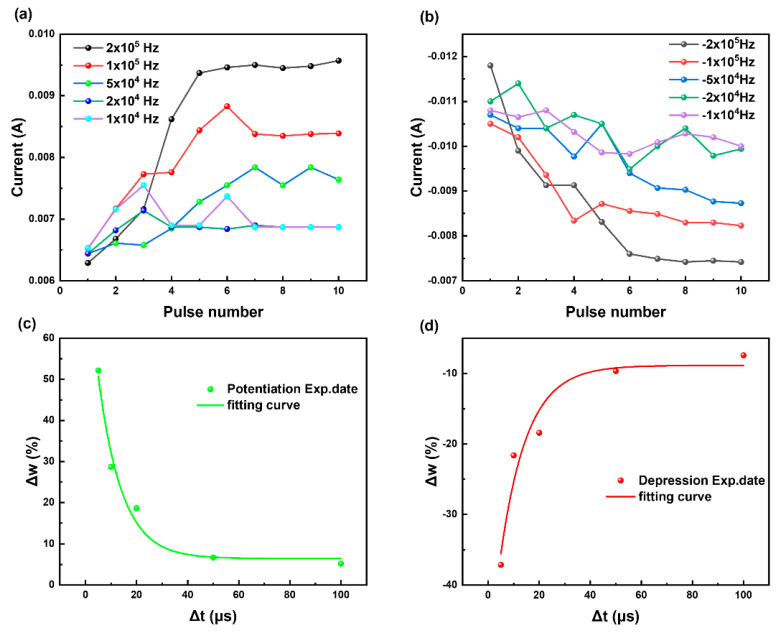
(**a**) Potentiation current curves from ten continuous pulses at Δ*t* = 5 μs, 10 μs, 20 μs, 50 μs, 100 μs; (**b**) depression current curves from ten continuous pulses at Δ*t* = 5 μs, 10 μs, 20 μs, 50 μs, 100 μs; (**c**) spike rate−dependent plasticity (SRDP) with potentiation; and (**d**) depression processes.

Simulations of PPF and PPD demonstrated excellent short-term synaptic plasticity of our device, as shown by the significant changes in its plasticity over short time intervals, which exponentially decreased upon increasing the interval. After clarifying the facilitation and suppression characteristics of the double pulse, we changed the double pulse signal to a continuous pulse signal and tested the device’s weight (Figures S6 and S7). When we applied continuous pulses of different frequencies to the device, the device weight also changed (Figure 5). The longer we applied a forward voltage pulse, i.e., the smaller the frequency of the pulse, the smaller the current increase of the device. Because a low-frequency positive pulse did not cause the continuous accumulation of active ions in the device, the device weight also decreased over time. The device weight was also obtained by continuously applying a high-frequency pulse wave. Similarly, we simulated a negative voltage pulse, and the test results were consistent with the positive voltage pulse. A core component of spatiotemporal information transmission is the response to a signal frequency [45]. Compared with signal amplitude, bionic memristors are more affected by the signal frequency, which corresponds to the basic characteristics of a neural system during the transmission of spatiotemporal information [46].

The Ebbinghaus forgetting curve was proposed by the German psychologist Ebbinghaus in 1885 and describes the relationship between memory and time:

$$G_{t}=G_{0}\times exp\left[-{\left(\frac{t}{\tau }\right)}^{\beta }\right]\;$$

where *G*_0_ and *G*_t_ are the device weight values, which correspond to the initial state of the memory and how it changes with time; t is time; τ is the relaxation time coefficient; β can change between 0 and 1. The weight retention law of synaptic bionic memristors should also conform to the Ebbinghaus amnesia curve, which is an important basis for testing the stability of a device. If no continuous strong pulse (high-amplitude, high-frequency pulse) is applied, the device weight values will decrease over time, just as human memory declines with time.

**Figure 6:**
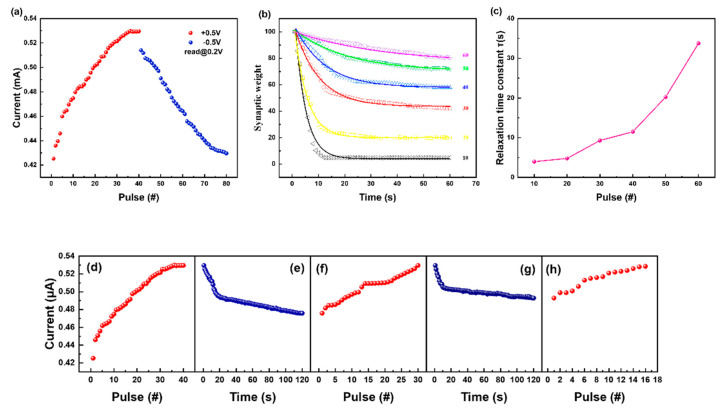
Simulated human brain memory behavior. (**a**) The current in response to a series of positive and negative voltage stimulations, showing the respective potentiation and depression of the device synaptic connection; (**b**) the retention curves for synaptic weight at different numbers of identical voltage pulse stimulations; (**c**) the relaxation time constant (τ) function of the number of stimulations; (**d**–**h**) demonstration of the learning/forgetting/relearning process. The amplitude, duration, and period of the voltage pulses were 0.5 V, 10 ms, and 1 s, respectively. The current responses were monitored using a low voltage of 0.2 V.

The brain’s memory function is achieved by spiking action potentials, and synaptic potential enhancement and inhibition are the basis of this function [47,48]. Therefore, we simulated the memory function of the human brain with the prepared memristor. The results showed that the weight of synapses, i.e., the magnitude of device current, was inhibited or enhanced when continuous negative or positive pulses were applied to the memristor. (Figure 6a). It is believed that there is a competitive relationship between the potentiation and inhibition processes of synaptic potentials during biological stimulation [49]. Figure 6b shows the synaptic weight retention curve, which rapidly decayed after applying a pulse and then gradually flattened. This result suggests that the number of identical voltage pulse stimuli with the same amplitude, period, and duration greatly affected the memory loss or retention of the device. Memory in humans mainly comes from short-term potential plasticity (STP) and long-term potential plasticity (LTP). As shown in Figure 6c, the time relaxation constant (τ) ranged from 4.05 s to 33.75 s as the number of pulse stimuli increased. This finding makes the transition from short-term memory to long-term memory possible in our memristor.

In this study, we explored the “learning/forgetting/relearning” process of the memristor (Figure 6d–h). When 40 consecutive voltage pulses were applied, the device current gradually increased with the number of pulses (Figure 6d). Compared with the magnitude of the current with memory function in humans, the observed “learning” processes were similar. Once the power supply was turned off, the device current gradually dropped to an intermediate state within 120 s (Figure 6e). This process represents “forgetting”. Then, to increase the device’s current to the magnitude of the first “learning” process, we only needed to stimulate the device with 30 consecutive pulses (Figure 6f). This “relearning” process is similar to the transition from STP to LTP, through which memory can be significantly enhanced. Next, we caused the device to go through the “forgetting” process a second time (Figure 6g). The device current at the end of the first “forgetting” process was 0.47 mA, while the device current at the end of the “re-forgetting” process reached 0.49 mA. This suggests that the speed of “remobilization” was slower than the speed of the first “forgetting” process. The process of applying the third pulse required only 16 voltage pulses, and the device current returned to the same level as at the end of the first learning period (Figure 6h). These results indicate that the voltage pulse applied to the device decreased over time. As the “relearning” process increased, fewer and fewer voltage pulses were applied to the device.

**Figure 7:**
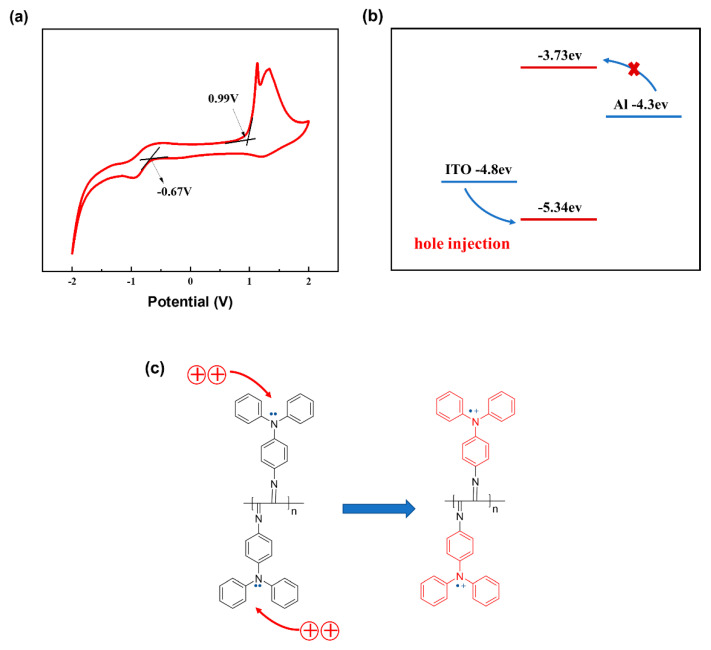
(**a**) Cyclic voltammograms of the PPIC film with an aqueous solution of tetrabutylammonium hexafluorophosphate (0.05 M) as the supporting electrolyte, Ag/AgCl as the reference electrode, and a scan rate of 100 mV/s; (**b**) the illustration of HOMO and LUMO energy levels of PPIC along with the work function of top/bottom electrodes (ITO and Al); (**c**) the redox−gated switching mechanism of the PPIC film device.

The main switching mechanisms proposed for devices based on organic/polymeric materials include redox interactions, charge transfer, conformational changes, and phase transitions [50]. Figure 7a shows the cyclic voltammetry curve of the PPIC film, with Ag/AgCl as the reference electrode. The initial oxidation and reduction potentials (E_ox_/E_red_) were +0.99 and −0.67 V, respectively. The HOMO/LUMO energy levels and bandgap values for PPIC were calculated using the following formula:

$$E_{HOMO/LUMO}=-\left[E_{onset\left(Ox./Red.vs.Ag/AgCl\right)}-E_{OX\left(ferrocene\right)}\right]-4.8$$

$$E_{g}=E_{LUMO}-E_{HUMO}$$

The HOMO, LUMO, and bandgap values of PPIC were calculated to be −5.34, −3.73, and 1.61 eV, respectively. The energy difference between the LUMO energy level of PPIC and the work function of Al (−4.3 eV) was found to be 0.57 eV. Similarly, the energy difference between the HOMO energy level of PPIC and the work function of ITO (−4.8 eV) was determined to be 0.54 eV. In comparison, the former value was smaller than the latter, suggesting that electron transport was the dominant factor in the conduction process of Al/PPIC/ITO. The TPA moiety has the capability to donate an electron, resulting in the formation of TPA^+^. Subsequently, TPA^+^ can accept an electron and revert back to its original state as TPA [51]. In the linear helical polymer PPIC, each unit exhibits redox properties, leading to unique electronic characteristics. When charge carriers are introduced from the electrode, they can travel along the polymer backbone plane via the delocalized π-electron system. The switching mechanism of the redox-active thin film involves a gradual transition between oxidized states, which occurs as the simulated voltage becomes more negative or positive. As illustrated, the effect of the accumulation of TPA^+^ moieties within the PPIC film on electronic transport can be seen when considering the example of negative voltage. Initially, the PPIC film without TPA^+^ moieties exhibits a high resistance to electronic charge carrier transport. However, after several rounds of negative voltage scanning, the active layer undergoes a reduction in resistance to electronic transport due to the formation and accumulation of TPA+ moieties. As the voltage scanning is further increased, the current value continues to rise until the changes between adjacent current values become indistinct, indicating the maximum accumulation of TPA^+^ moieties within the film. Consequently, the resistance of the PPIC region decreases during the voltage scanning process. When applying positive voltage scanning, the reverse evolution process of the oxidized state is observed. As the positive voltage simulation increases, the accumulation of TPA^+^ moieties within the PPIC film gradually decreases. The current value decreases with the increase in positive voltage scanning. Moreover, the quantity of TPA^+^ moieties with positive charge varies depending on the frequency and intensity of the applied voltage. The distinctive electrochemical properties establish the PPIC thin film as an exceptional memristive material.

## 4. Conclusions

In this paper, a helical polymer PPIC was synthesized via the polymerization of the N≡C group in 4-isocyano-*N*, *N*-diphenyl aniline and used to prepare a bionic memristor with a sandwich-layered structure. The flexible electronic device with an Al/PPIC/ITO-coated PET configuration showed good nonvolatile rewritable memory performance, even under repeated bending and tensile strain. Based on the synaptic function, key issues such as the perception and transmission of memristor processing spatiotemporal information were studied, and the influence of the pulse frequency on the device weight was larger. When the device weight was stimulated by a strong pulse, the device weight decayed slowly, and its retention time was longer, thus forming a more stable long-term memory. These findings indicate that helical PPIC fibers show promise in the development of flexible storage devices and biomimetic neural synapses. Additionally, the flexible memristor we created has the potential to integrate with flexible electronic systems and could potentially replace neural prosthetics, presenting innovative possibilities in the area of human-body biomimicry.

## Figures and Tables

**Table 1:** Voltage characteristics comparison with semiconductor memristors and our work of flexible memristors.

Active Layer	ON/OFF Ratio	ON (V)	OFF (V)	Ref.
CPTPP-NI	10^5^	1.70	−3.25	[29]
PBDTT-BQPy	10^3^	2.00	−1.20	[30]
PFTPA-AZO	10^2^	1.20	−1.00	[31]
MoS2-PDA-tBu4PcTiO	10^3^	2.85	−2.80	[32]
PPIC	10^3^	−0.72	1.79	This work

## Data Availability

Not applicable.

## Supplementary Materials

The following supporting information can be downloaded at: https://www.mdpi.com/article/10.3390/biomimetics8050391/s1, Figure S1: Synthetic route of PPIC. Figure S2: FTIR spectra of ICP and PPIC. Figure S3: FESEM image of the PPIC film. Figure S4: TGA, UV-vis absorption spectra and Photoluminescence spectra of the sample. Figure S5: AFM images of the samples. Figure S6: Ten consecutive pulses with a positive voltage. Figure S7: Ten consecutive pulses with a negative pulse.

## References

[1] Sun F., Lu Q., Feng S., Zhang T. (2021). Flexible artificial sensory systems based on neuromorphic devices. ACS Nano.

[2] Xu Y., Liu W., Huang Y., Jin C., Zhou B., Sun J., Yang J. (2021). Recent advances in flexible organic synaptic transistors. Adv. Electron. Mater..

[3] Ni Y., Han H., Liu J., Choi Y., Liu L., Xu Z., Yang L., Jiang C., Gao W., Xu W. (2022). A fibrous neuromorphic device for multi-level nerve pathways implementing knee jerk reflex and cognitive activities. Nano Energy.

[4] Zhu Y., Liang S., Mathayan V., Nyberg T., Primetzhofer D., Shi X., Zhang Z. (2022). High Performance Full-Inorganic Flexible Memristor with Combined Resistance-Switching. ACS Appl. Mater. Interfaces.

[5] Zhang H., Liu R., Zhao H., Sun Z., Liu Z., He L., Li Y. (2021). Research Progress of Biomimetic Memristor Flexible Synapse. Coatings.

[6] Jang B.C., Kim S., Yang Y., Park J., Cha H., Oh J., Choi Y. (2019). Polymer Analog Memristive Synapse with Atomic-Scale Conductive Filament for Flexible Neuromorphic Computing System. Nano Lett..

[7] Baeg K.J., Khim D., Kim J., Yang B.D., Kang M., Jung S.W., You I.K., Kim D.Y., Noh Y.Y. (2012). High-performance top-gated organic field-effect transistor memory using electrets for monolithic printed flexible NAND flash memory. Adv. Funct. Mater..

[8] Chen G., Fang Y., Zhao X., Tat T., Chen J. (2021). Textiles for learning tactile interactions. Nat. Electron..

[9] Bae H., Kim D., Seo M., Jin I.K., Jeon S.B., Lee H.M., Jung S.H., Jang B.C., Son G., Yu K. (2019). Bioinspired Polydopamine-Based Resistive-Switching Memory on Cotton Fabric for Wearable Neuromorphic Device Applications. Adv. Mater. Technol..

[10] Moin A., Zhou A., Rahimi A., Menon A., Benatti S., Alexandrov G., Tamakloe S., Ting J., Yamamoto N., Khan Y. (2021). A wearable biosensing system with in-sensor adaptive machine learning for hand gesture recognition. Nature Electronics..

[11] Zhou F., Chai Y. (2020). Near-sensor and in-sensor computing. Nat. Electron..

[12] Khan M., Rehman H.M.M.U., Tehreem R., Saqib M., Rehman M.M., Kim W.-Y. (2022). All-Printed Flexible Memristor with Metal–Non-Metal-Doped TiO2 Nanoparticle Thin Films. Nanomaterials.

[13] Lu Q., Sun F., Liu L., Li L., Wang Y., Hao M., Wang Z., Wang S., Zhang T. (2020). Biological receptor-inspired flexible artificial synapse based on ionic dynamics. Microsyst. Nanoeng..

[14] Xu T., Du H., Liu H., Liu W., Zhang X., Si C., Liu P., Zhang K. (2021). Advanced Nanocellulose-Based Composites for Flexible Functional Energy Storage Devices. Adv. Mater..

[15] Khot A.C., Dongale T.D., Park J.H., Kesavan A.V., Kim T.G. (2021). Ti3C2-based MXene oxide nanosheets for resistive memory and synaptic learning applications. ACS Appl. Mater. Interfaces.

[16] Zhao X., Wang Z., Xie Y., Xu H., Zhu J., Zhang X., Liu W., Yang G., Ma J., Liu Y. (2018). Photocatalytic reduction of graphene oxide-TiO2 nanocomposites for improving resistive-switching memory behaviors. Small.

[17] Park S., Liao Z., Ibarlucea B., Qi H., Lin H.-H., Becker D., Melidonie J., Zhang T., Sahabudeen H., Baraban L. (2020). Two-dimensional boronate ester covalent organic framework thin films with large single crystalline domains for a neuromorphic memory device. Angew. Chem. Int. Ed..

[18] Lee T., Lee W., Kim S.W., Kim J.J., Kim B.S. (2016). Flexible textile strain wireless sensor functionalized with hybrid carbon nanomaterials supported ZnO nanowires with controlled aspect ratio. Adv. Funct. Mater..

[19] Younis A., Chu D., Lin X., Yi J., Dang F., Li S. (2013). High-performance nanocomposite based memristor with controlled quantum dots as charge traps. ACS Appl. Mater. Interfaces.

[20] Zheng G., Cui Y., Karabulut E., Wagberg L., Zhu H., Hu L. (2013). Nanostructured paper for flexible energy and electronic devices. MRS Bull..

[21] Zhang Y., Zhang L., Cui K., Ge S., Cheng X., Yan M., Liu H. (2018). Flexible Electronics Based on Micro/Nanostructured Paper. Adv. Mater..

[22] Hirst A.R., Escuder B., Miravet J.F., Smith D.K. (2008). High-Tech Applications of Self-Assembling Supramolecular Nanostructured Gel-Phase Materials: From Regenerative Medicine to Electronic Devices. Angew. Chem. Int. Ed..

[23] Wang D., Zhao S., Li L., Wang L., Cui S., Wang S., Shen G. (2022). All-Flexible Artificial Reflex Arc Based on Threshold-Switching Memristor. Adv. Funct. Mater..

[24] Wang Y., Meng L., Rao Y., He Y., Chen L., Zhu H., Zhang W. (2020). Three-Dimensional Nanoscale Flexible Memristor Networks with Ultralow Power for Information Transmission and Processing Application. Nano Lett..

[25] Roncali J., Leriche P., Blanchard P. (2014). Molecular materials for organic photovoltaics: Small is beautiful. Adv. Mater..

[26] Liu C., Chen W. (2011). Donor–acceptor polymers for advanced memory device applications. Polym. Chem..

[27] Zhang B., Chen Y., Liu G., Xu L.-Q., Chen J., Zhu C.-X., Neoh K., Kang E.-T. (2012). Push–Pull archetype of reduced graphene oxide functionalized with polyfluorene for nonvolatile rewritable memory. J. Polym. Sci. Part A Polym. Chem..

[28] Roncali J. (2009). Molecular bulk heterojunctions: An emerging approach to organic solar cells. Acc. Chem. Res..

[29] Xiao X., Zhou F., Jiang J., Chen H., Wang L., Chen D., Lu J. (2018). Highly efficient polymerization via sulfur(vi)-fluoride exchange (SuFEx): Novel polysulfates bearing a pyrazoline–naphthylamide conjugated moiety and their electrical memory performance. Polym. Chem..

[30] Sun J., He Z., Liu S., Fan F., Chen W., Zhang B., Liu G. (2021). Intramolecular rotation induced High-Temperature Self-Optimization for polymer memristor devices. Eur. Polym. J..

[31] Sun J., Chen Q., Fan F., Zhang Z., Han T., He Z., Liu G. (2022). A dual-mode organic memristor for coordinated visual perceptive computing. Fundam. Res..

[32] Yan Q., Fan F., Sun C., El-Khouly M.E., Liu H., Zheng Y., Zhang B., Liu G., Chen Y. (2021). MoS2 nanosheets chemically modified with metal phthalocyanine via mussel-inspired chemistry for multifunctional memristive devices. J. Mater. Chem. C.

[33] Brunsveld L., Folmer B., Meijer E., Sijbesma R. (2001). Supramolecular polymers. Chem. Rev..

[34] Liu Y., He K., Chen G., Leow W.R., Chen X. (2017). Nature-inspired structural materials for flexible electronic devices. Chem. Rev..

[35] Choi S., Lee H., Ghaffari R., Hyeon T., Kim D. (2016). Recent advances in flexible and stretchable bio-electronic devices integrated with nanomaterials. Adv. Mater..

[36] Xia Y., He Y., Zhang F., Liu Y., Leng J. (2021). A review of shape memory polymers and composites: Mechanisms, materials, and applications. Adv. Mater..

[37] Baeg K.-J., Noh Y.-Y., Sirringhaus H., Kim D.-Y. (2010). Controllable shifts in threshold voltage of top-gate polymer field-effect transistors for applications in organic nano floating gate memory. Adv. Funct. Mater..

[38] Tononi G., Cirelli C. (2014). Sleep and the price of plasticity: From synaptic and cellular homeostasis to memory consolidation and integration. Neuron.

[39] Stein R.B., Gossen E.R., Jones K.E. (2005). Neuronal variability: Noise or part of the signal. Nat. Rev. Neurosci..

[40] Pulido C., Marty A. (2017). Quantal fluctuations in central mammalian synapses: Functional role of vesicular docking sites. Physiol. Rev..

[41] Yoshihara M., Littleton J.T. (2002). Synaptotagmin I functions as a calcium sensor to synchronize neurotransmitter release. Neuron.

[42] Wang Y., Yin L., Huang W., Li Y., Huang S., Zhu Y., Yang D., Pi X. (2021). Optoelectronic synaptic devices for neuromorphic computing. Adv. Intell. Syst..

[43] Dolphin A.C., Lee A. (2020). Presynaptic calcium channels: Specialized control of synaptic neurotransmitter release. Nat. Rev. Neurosci..

[44] Cao G., Meng P., Chen J., Liu H., Bian R., Zhu C., Liu F., Liu Z. (2021). 2D material based synaptic devices for neuromorphic computing. Adv. Funct. Mater..

[45] He Y., Nie S., Liu R., Jiang S., Shi Y., Wan Q. (2019). Spatiotemporal Information Processing Emulated by Multiterminal Neuro-Transistor. Adv. Mater..

[46] Chen Y., Lu Y., Liao M., Tian Y., Liu Q., Gao C., Yang X., Shan C. (2019). 3D Solar-Blind Ga_2_O_3_ Photodetector Array Realized Via Origami Method. Adv. Funct. Mater..

[47] Dolphin A.C., Lee A. (2009). Amyloid-beta as a positive endogenous regulator of release probability at hippocampal synapses. Nat. Rev. Neurosci..

[48] Shu Y., Hasenstaub A., Duque A., Yu Y., McCormick D.A. (2006). Modulation of intracortical synaptic potentials by presynaptic somatic membrane potential. Nature.

[49] Rossum M., Bi G., Turrigiano G. (2000). Stable Hebbian learning from spike timing-dependent plasticity. J. Neurosci..

[50] Ling D., Liaw J., Teo H., Zhu C., Chan H., Kang T., Neoh G. (2007). Polymer memories: Bistable electrical switching and device performance. Polymer.

[51] Wang X., Tang W., Loh P. (2021). Para-Substituted Triphenylamine as a Catholyte for Zinc-Organic Aqueous Redox Flow Batteries. ACS Appl. Energy Mater..

