# Supplementary material for: Triphenylamine-Based Helical Polymer for Flexible Memristors
Source: Biomimetics (Basel). 2023 Aug 26;8(5):391. doi: 10.3390/biomimetics8050391 (PMC10526500; doi:10.3390/biomimetics8050391)
Supplement: Supplementary file 1 [file biomimetics-08-00391-s001.zip › biomimetics-2553035-supplementary.pdf]

Supporting Information

Triphenylamine-Based Helical Polymer for Flexible Memristors

Jinyong Li [1], Minglei Gong [2], Xiaoyang Wang [3], Fei Fan [2,*] and Bin Zhang [1,*]

[1] Key Laboratory for Advanced Materials and Joint International Research Laboratory of Precision Chemistry and Molecular Engineering, School of Chemistry and Molecular Engineering, East China University of Science and Technology, Shanghai (200237), China

[2] Shanghai i-Reader Biotech Co., Ltd., Shanghai (201100), China

[3] Guangxi Key Laboratory of Information Material, Engineering Research Center of Electronic Information Materials and Devices, School of Material Science and Engineering, Guilin University of Electronic Technology, Guilin (541200), China

* Correspondence: fanfei@i-reader.cn (F.F.); zhangbin@ecust.edu.cn (B.Z.)

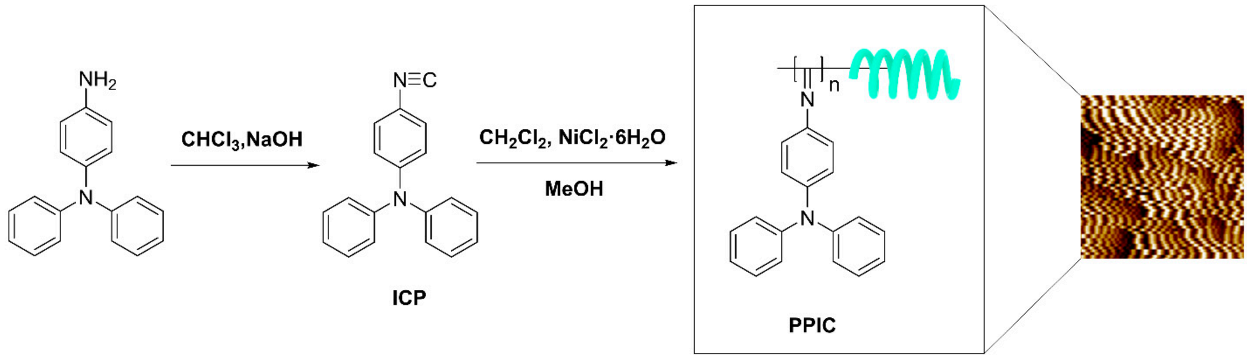

Figure S1 Synthetic route of PPIC.

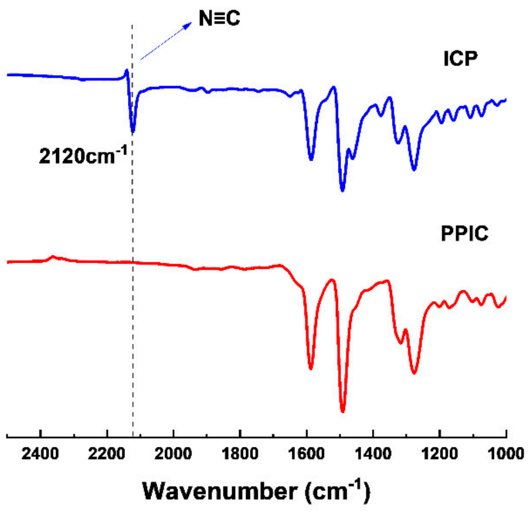

Figure S2 FTIR spectra of ICP and PPIC.

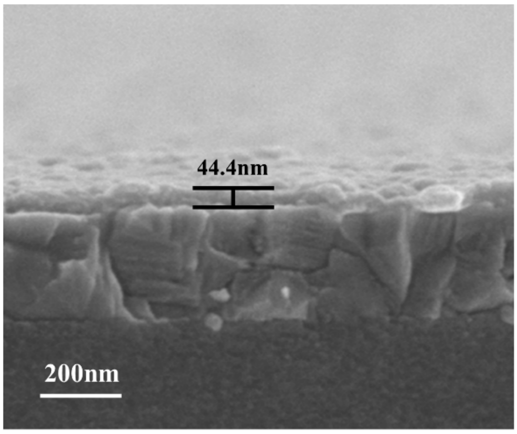

Figure S3 FESEM image of the PPIC film.

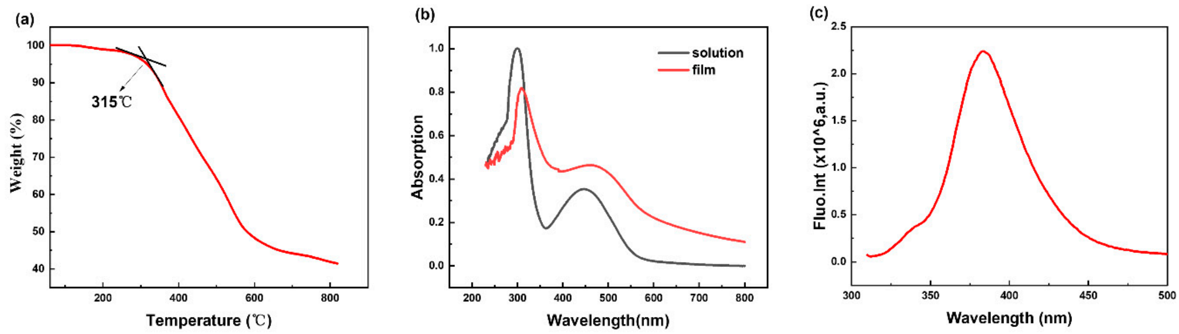

Figure S4 (a) TGA curve of PPIC; (b) UV-vis absorption spectra of the PPIC solution and film; (c) Photoluminescence spectra of PPIC.

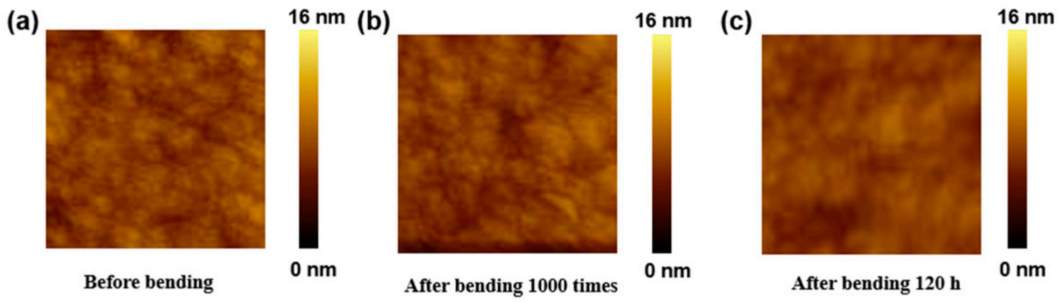

Figure S5 AFM image (2 x 2 μm) of PPIC thin-films coated on PET (a) before bending; (b) after bending 1000 times; (C) after bending for 120 h.

To investigate the flexibility of PPIC, we characterized its morphology and structure by AFM, which showed that the roughness of the film did not change significantly after bending 1000 times or for 120 h.

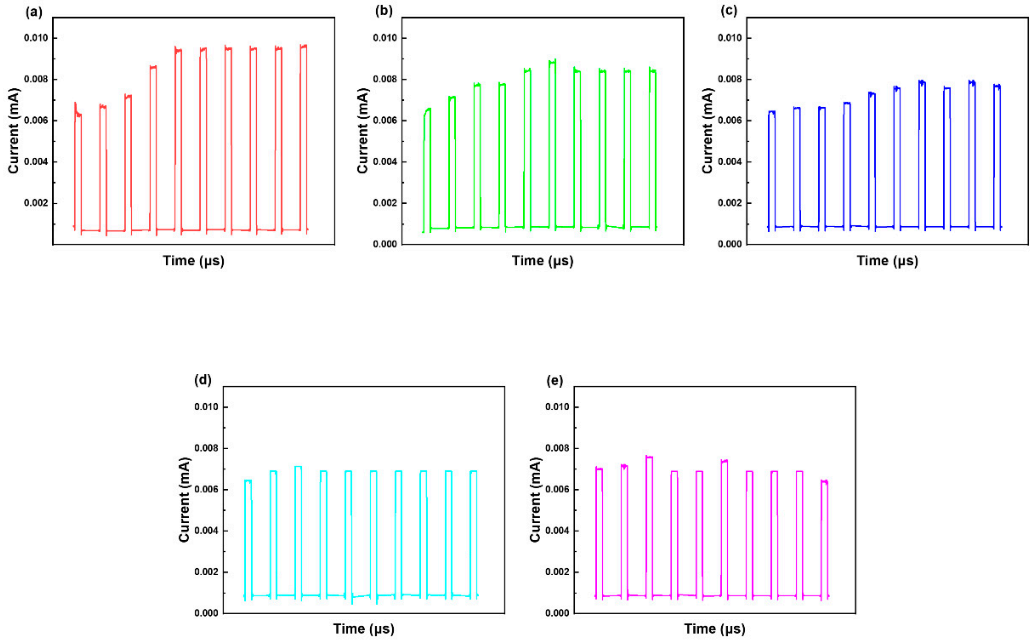

Figure S6 a, b, c, d, e Ten consecutive pulses with a positive voltage (amplitude 2 V, pulse width 30 μs, Δt =5 μs, 10 μs, 20 μs, 50 μs, 100 μs)

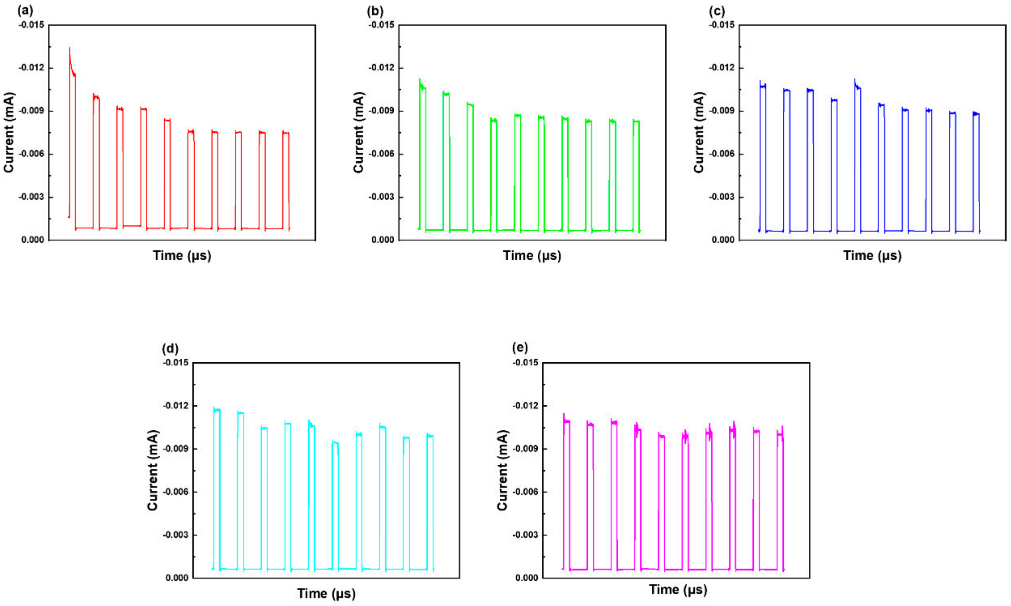

Figure S7 a, b, c, d, e Ten consecutive pulses with a negative pulse (amplitude -2 V, pulse width 30 μs, Δt = 5 μs, 10 μs, 20 μs, 50 μs, 100 μs).
